# STAT1 regulates neutrophil gelatinase B-associated lipocalin induction in influenza-induced myocarditis

**DOI:** 10.1038/s41598-024-61953-z

**Published:** 2024-05-15

**Authors:** Nicholas J. Constantinesco, Sashwath Srikanth, Louis De Vito, Crystal Moras, Vennila Ramasubramanian, Baskaran Chinnappan, Sean Hartwick, Kristina E. Schwab, Yijen Wu, Radha Gopal

**Affiliations:** Department of Pediatrics, UPMC Children’s Hospital of Pittsburgh, University of Pittsburgh, Pittsburgh, PA USA

**Keywords:** Immunology, Microbiology, Cardiology, Diseases, Medical research

## Abstract

Influenza is a significant public health and economic threat around the world. Epidemiological studies have demonstrated a close association between influenza pandemics and cardiovascular mortality. Moreover, it has been shown that there is a decrease in cardiovascular mortality in high-risk patients following vaccination with the influenza vaccine. Here, we have investigated the role of anti-viral STAT1 signaling in influenza-induced myocarditis. Wild-type mice (C57BL/6) were infected with either influenza A/PR/8/34 or control, and cellular response and gene expression analysis from the heart samples were assessed 7 days later. The expression of interferon response genes STAT1, STAT2, Mx1, OASL2, ISG15, chemokines CCL2, CCL3, CXCL9 and CXCL10, and the frequency of neutrophils (CD45^+^CD11b^+^Ly6G^+^) and CD4+ T cells (CD45^+^CD4^+^) were all significantly increased in influenza-infected mice when compared to vehicle controls. These data suggest that influenza infection induces interferons, inflammatory chemokines, and cellular recruitment during influenza infection. We further investigated the role of STAT1 in influenza-induced myocarditis. The frequency of neutrophils and the levels of lipocalin 2 were significantly increased in STAT1^−/−^ mice when compared to WT controls. Finally, we investigated the role of Lcn2 in viral-induced myocarditis. We found that in the absence of Lcn2, there was preserved cardiac function in Lcn2^−/−^ mice when compared to WT controls. These data suggest that the absence of Lcn2 is cardioprotective during viral-induced myocarditis.

## Introduction

Influenza is a significant worldwide public health and economic threat. It is estimated that 3–4 million cases of severe illness and 300,000 deaths due to influenza infection occur annually. Pandemic outbreaks of highly virulent strains of influenza can have a major impact on healthcare settings. During influenza pandemics, lung disease, the most common cause of death, is a primary focus. However, epidemiological studies have indicated that influenza seasons positively correlate with increased numbers of hospitalizations and mortality due to cardiovascular diseases^1–4^. Further, studies have shown that influenza vaccination significantly reduces the risk of cardiovascular mortality^5–7^. In the 2009 pandemic, it has been estimated that influenza A/pH1N1 caused 201,200 respiratory deaths and 83,300 cardiovascular deaths^8^. Clinical reports show that acute myocarditis, cardiomyopathy in adults, and fulminant myocarditis in children occurred during the 2009 H1N1 pandemic^9–11^. Several cases of influenza-associated myocarditis showed elevation in cardiac injury markers and abnormal ECG findings^12,13^. However, the pathogenic mechanism involved in influenza-induced myocarditis is not clear.

During viral infection, the innate and adaptive immune systems activate a variety of immune signaling pathways that induce type I (IFNα/β), type II (IFNγ), and type III (IFNλ) interferons (IFNs), and a variety of inflammatory cytokines and chemokines^14–17^. IFNs inhibit viral replication by inducing interferon-stimulated genes (ISGs), such as MX dynamin-like GTPase1 (Mx1) and 2ʹ-5ʹ-Oligoadenylate synthase-like protein (OASL2), through Janus kinase (JNK)-dependent phosphorylation of Stat1 and Stat2^18^. IFNs and inflammatory mediators recruit monocytes, neutrophils, and macrophages to the lungs for viral control. However, the excessive influx of innate immune cells and the dysregulated production of inflammatory cytokines result in host-mediated pathological responses during viral infection^14,17,19–21^.

In the heart, viral infection-associated disease can occur by either direct viral entry or due to the systemic inflammatory response initiated in the lung. Studies have shown that influenza virus infection induces host cell proteases, such as trypsin and matrix metalloprotease 9 (MMP-9) in various organs, which may cause increased vascular permeability and disease propagation in the myocardium^22,23^. Further, a recent study has shown influenza viral replication in cardiomyocytes and Purkinje cells in mice^24^.

Neutrophil gelatinase-associated lipocalin (NGAL) or Lcn2 is a secreted 25 kDa protein. Studies have shown that Lcn2 plays a role in iron trafficking and has chemotactic and bacteriostatic properties^25–27^. It has also been used as a biomarker for acute kidney injury because of its release due to tubular damage. Recently, it has been shown that Lcn2 is induced in coronary heart diseases, heart failure, and myocarditis and plays a role in the pathophysiology of cardiovascular diseases (CVD)^28,29^.

In this study, we established the murine model of immune responses to influenza infection in the heart. Influenza infection increased the expression of interferon response genes as well as proinflammatory cytokines and chemokines, and increased frequency of neutrophils. Further, we analyzed the role of Stat1 in the heart during influenza infection. We found an increased frequency of neutrophils and increased expression of Lcn2 in the heart in Stat1^−/−^ mice when compared to WT mice. These findings establish a potential mechanism by which interferon signaling through Stat1 suppresses the inflammatory immune response in the heart during influenza infection.

## Results

### Influenza infection increases the expression of interferon-stimulated genes (ISGs), proinflammatory chemokines, and the frequency of neutrophils in the heart

**Figure 1:**
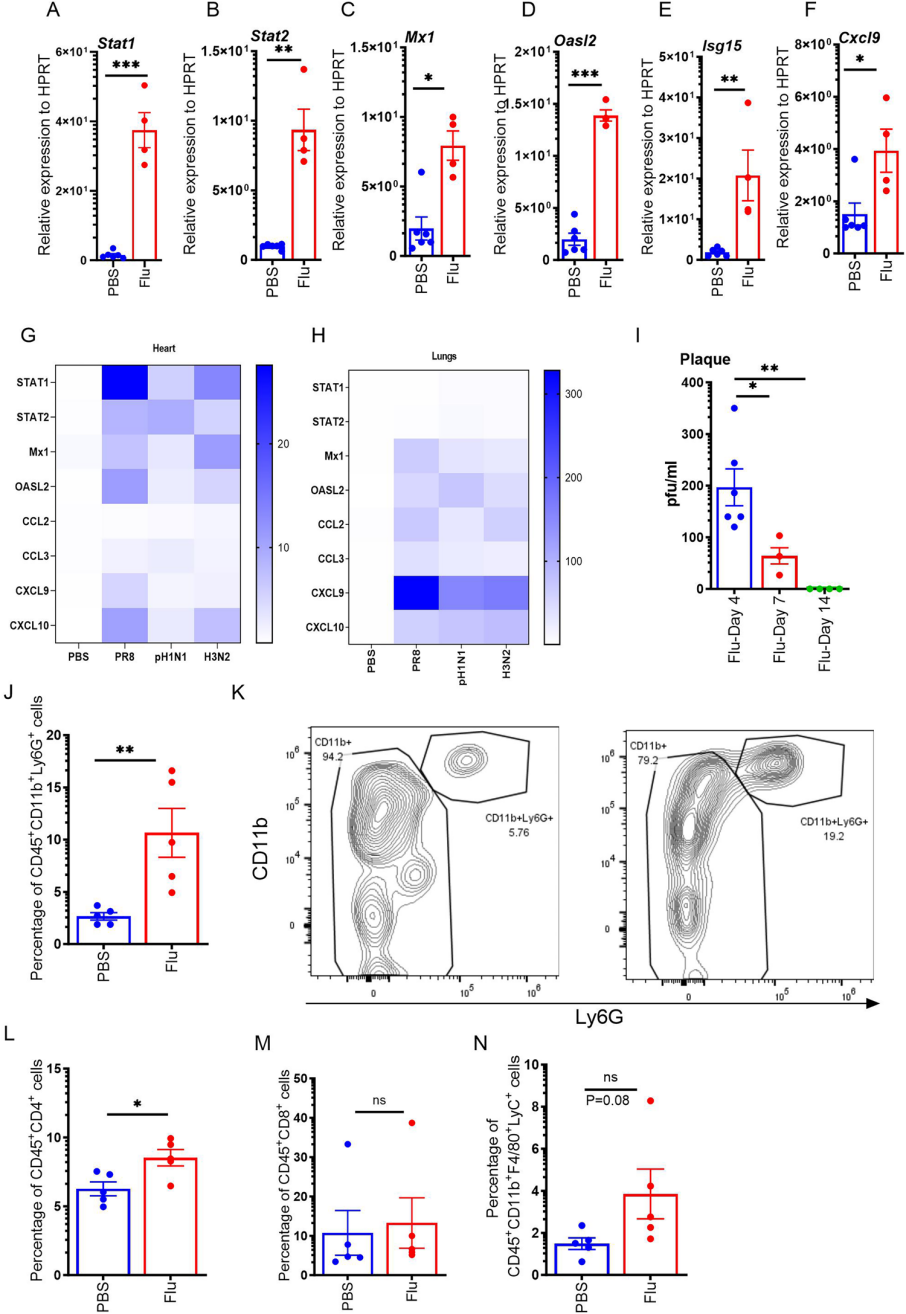
Influenza infection increases the expression of interferon-stimulated genes (ISGs), proinflammatory chemokines, and the frequency of neutrophils and lymphocytes in the heart. C57BL/6 mice were treated with PBS or 10^3^ PFU of influenza A PR/8/34 on day 0, left for 7 days, and sacrificed on day 7. Relative expressions of STAT1, STAT2, Mx1, OASL2, ISG15, and CXCL9 in the hearts were measured by RT-PCR (n = 4–6) (**A–F**). Stat1, Stat2, Mx1, Oasl2, CCL2, CCL3, CXCL9, and CXCL10 were analyzed from PR8, pH1N1, and H3N2 strains of influenza from the heart (n = 5–7) (**G**) and the lungs (n = 5–7) (**H**). A plaque assay was carried out from heart sample homogenates (n = 4–6) (**I**). The percentage of CD45^+^CD11b^+^Ly6G^+^ cells in the heart was measured by flow cytometry (n = 5) (**J**). Representative flow cytometry plots were shown (**K**). The percentage of CD45^+^CD4^+^T cells, CD45^+^CD8^+^T cells, and CD45^+^CD11b^+^F4/80^+^Ly6C^+^ cells in the heart were measured by flow cytometry (n = 5) (**L–N**). Data are represented as mean ± SEM. Significance was tested by unpaired t-test or one-way ANOVA. *p < 0.05, **p < 0.01, *ns* not significant. Each experiment was independently performed two or more times, and the representative data is shown.

During early infection, the influenza virus induces the expression of type I and type III interferons, and pro-inflammatory cytokines, while also inducing cellular recruitment in the lung for viral control. Studies have shown an increased incidence rate of cardiovascular events during influenza season. However, the pathogenesis due to influenza-induced myocarditis is not clear. To determine the effect of influenza in the heart, we infected C57BL/6 mice with influenza A/PR/8/34 H1N1 or a PBS control and measured the interferon response genes STAT1, STAT2, Mx1 (MX Dynamin Like GTPase 1), OASL2 (2'-5'-Oligoadenylate Synthetase Like), ISG15 (Interferon stimulated gene-15), and CXCL9 on day 7 after influenza viral treatment. We observed increased expression of all these genes in the influenza-infected mice when compared to PBS-treated controls (Fig. 1A–F). These data suggest that influenza infection induces interferon responses in the heart.

Next, we analyzed the expression of interferon response genes Stat1, Stat2, Mx1, Oasl2, and proinflammatory immune response genes CCL2, CCL3, CXCL9, and CXCL10 from influenza A/PR/8/34 H1N1, pandemic H1N1, and seasonal H3N2, strains of influenza viruses in the heart and compared them with lung responses. We observed increased expression of Stat1, Stat2, Mx1, Oasl2, CCL2, CCL3, CXCL9, and CXCL10 in influenza-infected hearts and lungs when compared to PBS-treated controls (Fig. 1G,H). The relative fold inductions were approximately tenfold in the heart when compared to expression levels in the 100-fold range in the lungs. These data suggest that the influenza virus infects the heart and induces interferon responses regardless of influenza strains.

Next, to determine if the virus translocated to the heart, we homogenized the hearts from mice on days 4, 7, and 14 following influenza infection. We found measurable viral titers on day 4 and day 7 after influenza infection (Fig. 1I). However, we found no viral titer in the hearts on day 14 following influenza infection (Fig. 1I). These data suggest that the influenza virus directly infects the heart when infected via a pulmonary route.

Next, we analyzed the cellular responses in mice treated with influenza and found that there was a higher frequency of CD45+ CD11b+ Ly6G+ neutrophils and CD45+ CD4+ T cells present in the hearts of influenza-infected mice when compared to the PBS controls (Fig. 1J–L). However, we found no significant differences in the frequency of CD45+ CD8+ T cells between influenza-infected mice and PBS controls (Fig. 1M). Next, we analyzed the frequency of CD45+ CD11b+ F4/80+ Ly6C+ cells or inflammatory monocytes and found that the frequency was trending to increase in influenza-infected hearts when compared to PBS control hearts (Fig. 1N). These data suggest that the virus infiltrated and managed to infect the heart and induce an inflammatory immune response.

**Figure 2:**
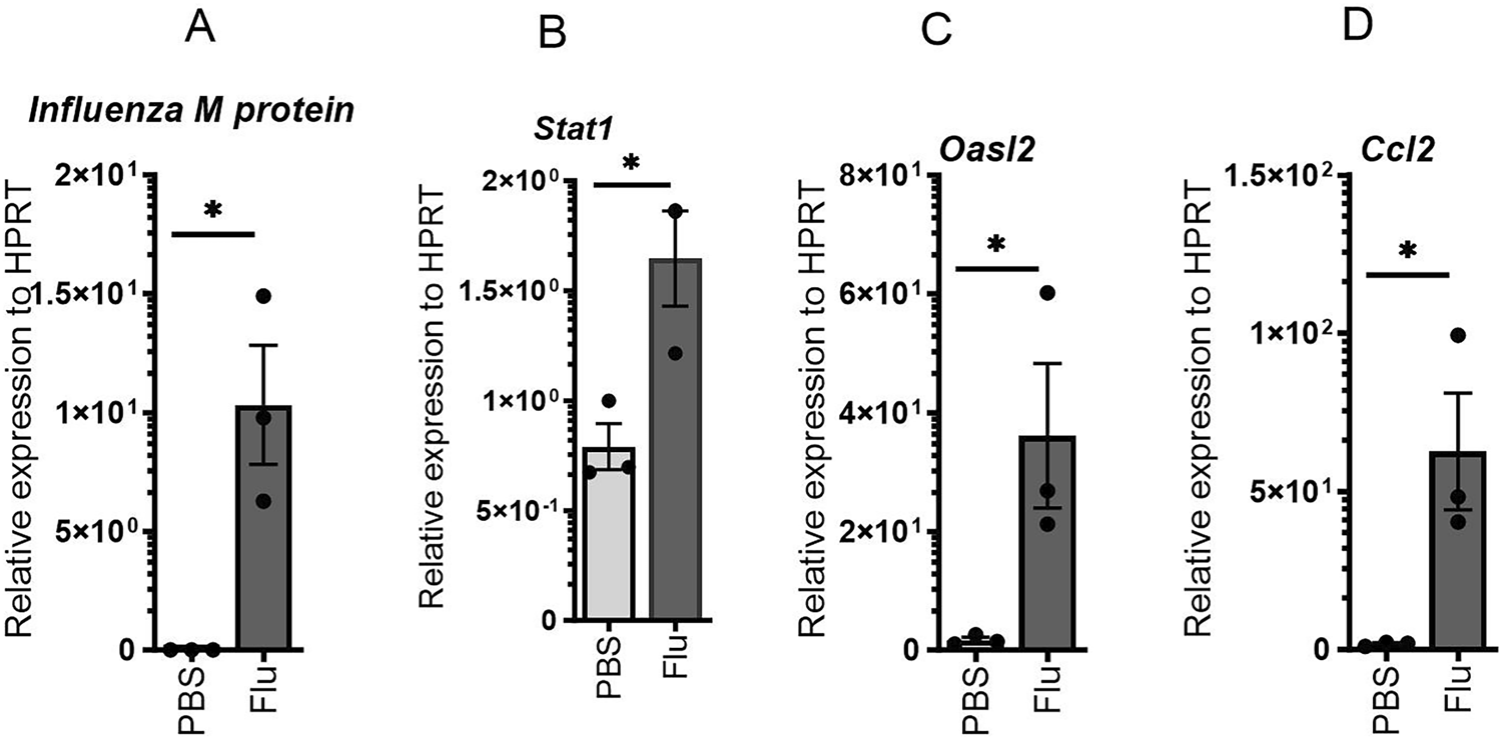
Influenza infection increases the expression of interferon response genes and proinflammatory chemokines in cardiomyocytes. Cardiomyocytes were isolated from C57BL/6 mice, and treated with influenza (1 MOI), and expression of Influenza M protein, Stat1, Oasl2, and CCL2 were analyzed by RT-PCR (n = 3). Data are represented as mean ± SEM. Significance was tested by unpaired t-test or one-way ANOVA. *p < 0.05, **p < 0.01, *ns* not significant. Each experiment was independently performed two or more times, and the representative data is shown.

Next, we wanted to see whether the influenza virus infects cardiomyocytes directly and induces interferon response genes and pro-inflammatory cytokines. We isolated cardiomyocytes from WT mice and placed them in media for 24 h. The cells were treated with PBS or influenza virus (MOI = 1) for 24 h. The cells were analyzed for interferon response genes and proinflammatory immune response. We found increased expression of influenza M protein, Stat1, Oasl2, and CCL2 in influenza-infected mice when compared to PBS-treated controls (Fig. 2A–D). These data suggest that the heart does signal for chemotaxis to the site of infection which could produce an inflammatory immune response. Furthermore, the increased expression of Stat1 and OASL2 suggests that the cardiomyocytes enter an antiviral state in the presence of the influenza virus, further demonstrating the ability of the virus to infect the cells and induce IFN responses in the heart.

### Influenza infection decreases left ventricular ejection fraction (LVEF) following influenza infection

**Figure 3:**
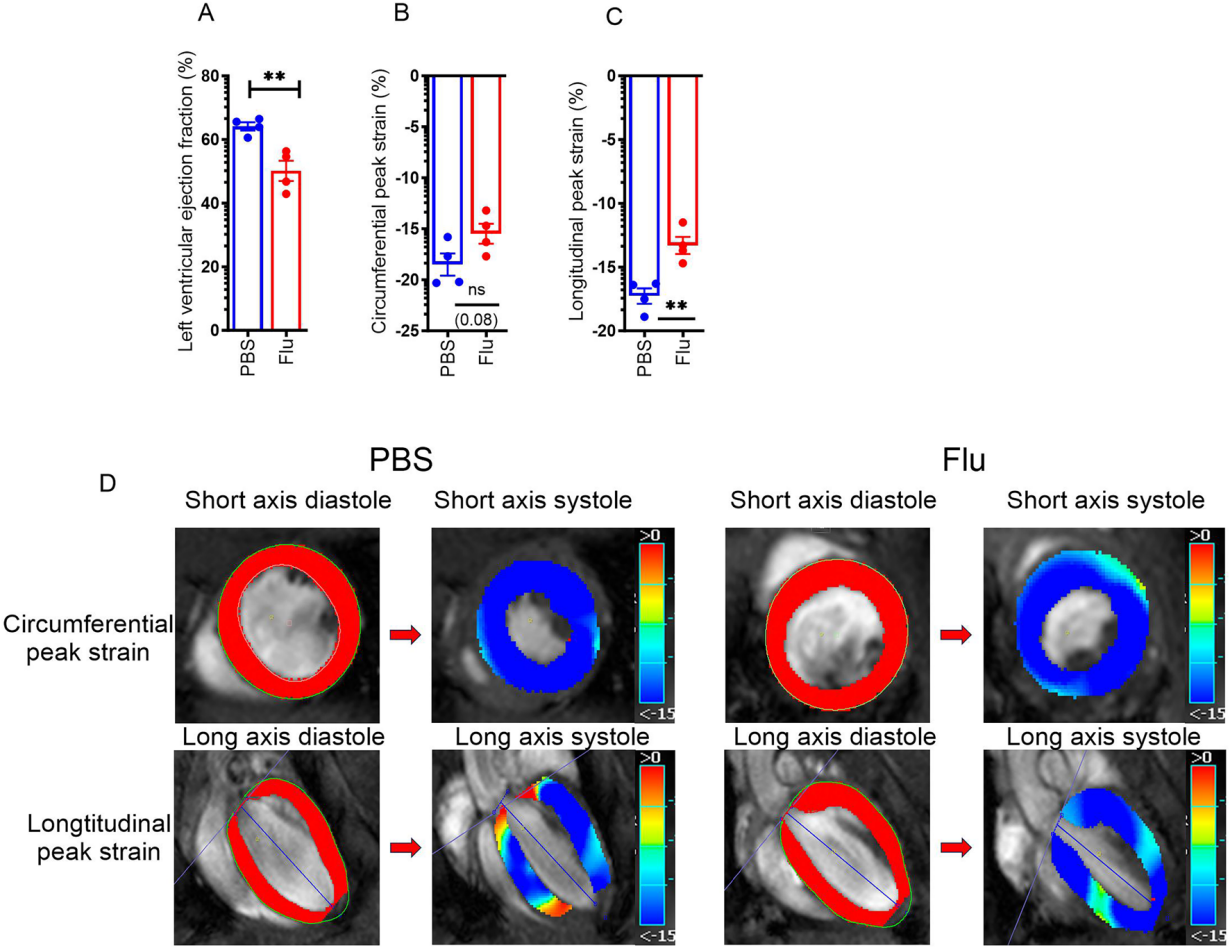
Influenza infection impairs heart functions during influenza-induced myocarditis**.** C57BL/6 mice were treated with PBS or 10^3^ PFU of influenza A PR/8/34 on day 0, left for 7 days, and sacrificed on day 7. The percentage of left ventricle ejection fraction (LVEF) (n = 4) (**A**), percentage of circumferential peak strain (n = 4) (**B**), and percentage of longitudinal peak strain (n = 4) (**C**) were analyzed from micro-MRI live imaging from C57BL/6 mice treated with PBS or influenza. The representative images are shown (**D**). The top row shows circumferential strain overlayed on the short-axis mid-slice cardiac cycle. The bottom row shows longitudinal strain overlayed on the long axis 4 chamber cardiac cycle. The blue line represents the long axis extent that runs from the aorta to the apex of the heart to calculate the LV function parameters (**D**). Data are represented as mean ± SEM. Significance was tested by unpaired t-test or one-way ANOVA. *p < 0.05, **p < 0.01, *ns* not significant. Each experiment was independently performed two or more times, and the representative data is shown.

Next, we determined whether the influenza-induced inflammatory immune response has an impact on heart function via in-vivo cardiac imaging by MRI. Mice were treated with PBS or influenza and the left ventricular ejection fraction (LVEF), circumferential, and longitudinal peak strains were measured on day 7 following influenza infection. We found that the LVEF was significantly decreased in influenza-treated mice when compared to the PBS controls (Fig. 3A). The LVEF values decreased from 66 to 42% which is indicative of mild myocarditis. Next, we found the circumferential peak strain was worse (− 20 to − 13) in influenza-infected mice when compared to PBS controls (Fig. 3B–D). The longitudinal peak strain was significantly impaired (− 16 to − 11) in influenza-infected mice when compared to PBS controls (Fig. 3C,D). These data suggest that heart function was compromised during pulmonary influenza infection.

### STAT1 regulates neutrophil infiltration in the heart during influenza infection

Studies have shown that STAT1 is crucial in the type I, II, and III IFN signaling pathways. After discovering STAT1 is highly expressed in the hearts of influenza-infected mice, we decided to investigate the role of STAT1 in the pathogenesis of myocarditis. First, we analyzed the response of ISGs to the elimination of STAT1 expression using STAT1^−/−^ mice. We infected both C57BL/6 and STAT1^−/−^ mice with influenza or PBS control and then measured the relative expression of Mx1 and the chemokine CXCL10. As expected, the expressions of Mx1 and CXCL10 were decreased in STAT1^−/−^ mice (Supplementary Fig. 1A,B). These data suggest that Stat1 is crucial in the induction of ISGs in the heart during influenza infection.

Next, we determined whether Stat1 controls the viral burden. We measured the viral burden and found that the viral burden was significantly increased in Stat1^−/−^ mice when compared to WT mice (Supplementary Fig. 2). These data suggest that Stat1 controls the viral burden in the heart.

Our previous studies suggested that STAT1 regulates Th17 responses during influenza infection^30^. Therefore, we measured the expression of IL-17 and IL-22 responses from the heart samples from WT and Stat^−/−^ mice. We found no differences in the expressions of IL-17RA, IL-17F, IL-22RA1, and IL-22RA2 and the levels of IL-17A in the heart samples from WT and Stat1^−/−^ mice (Supplementary Fig. 3A–E). These data suggest that Stat1 does not regulate the IL-17 responses in influenza-induced myocarditis.

**Figure 4:**
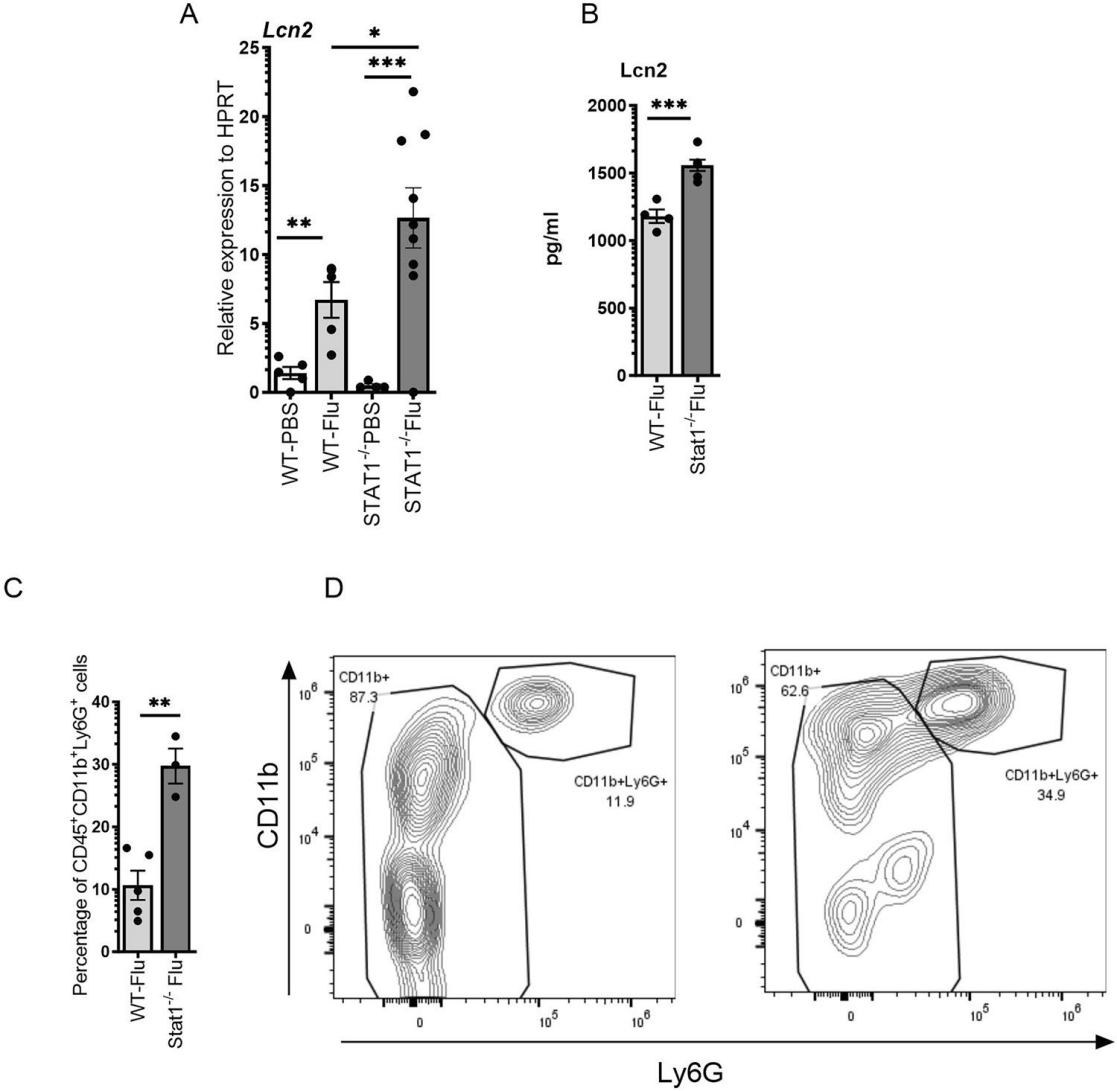
The elimination of STAT1 expression suppresses the neutrophils. WT or STAT1^−/−^ mice were treated with PBS or 10^3^ PFU of influenza A PR/8/34 on day 0, left for 7 days, and sacrificed on day 7. Relative expression of Lcn2 was carried out from WT and Stat^−/−^ mice (n = 4–9) (**A**). The Lcn2 protein levels were measured from WT and Stat1^−/−^ mice (n = 4–6) (**B**). The percentage of CD45^+^CD11b^+^Ly6G^+^ cells in the hearts of STAT1^−/−^ mice was measured via flow cytometry (n = 3–5) (**C**). Representative flow cytometry plots are shown (**D**). Data are represented as mean ± SEM. Significance was tested by unpaired t-test or one-way ANOVA. *p < 0.05, **p < 0.01, *ns* not significant. Each experiment was independently performed two or more times, and the representative data is shown.

Lipocalin 2 is an antimicrobial peptide produced in response to inflammatory cytokines, especially IL-17. Our studies have shown that IL-17 and TNFα synergistically induce Lcn2^31^. Therefore, we determined whether STAT1 affects Lcn2 expression in influenza-induced myocarditis. We found the Lcn2 expression was significantly increased in the hearts from Stat1^−/−^ when compared to WT mice during influenza infection (Fig. 4A). We further measured the Lcn2 protein levels and found the levels were increased in the hearts of influenza-infected Stat1^−/−^ mice when compared to influenza-infected WT mice (Fig. 4B). These data suggest that STAT1 plays a role in regulating the expression of Lcn2 in influenza-induced myocarditis.

Following influenza infection, the innate immune response induces pro-inflammatory cytokines and neutrophil recruitment. The neutrophil chemoattractant CXCL1, CXCL2, and CXCL5 increase recruitment to promote viral control. Previously we have shown that the STAT1 antigen-presenting cells were biased towards Th17 response. Th17 response is known to drive neutrophilic immune responses, therefore, we measured whether Stat1 has any effect on neutrophilic responses. We found that there was a significantly higher frequency of CD45+ CD11b+ Ly6G+ neutrophils present in the hearts of STAT1^−/−^ mice infected with influenza compared to the WT control mice (Fig. 4C,D). These data suggest that Stat1 suppresses the neutrophilic infiltration in influenza-induced myocarditis.

Next, we conducted an MRI scan measuring the LVEF, circumferential, and longitudinal strain from WT and Stat1^−/−^ mice infected with influenza. We found no significant differences between WT and STAT1^−/−^ in LVEF, circumferential and longitudinal strains (Supplementary Fig. 4A–D). These data suggest that despite Stat1 regulation of neutrophilic infiltration it does not impact the heart function during pulmonary influenza infection.

### Suppression of Lcn2 expression improves outcomes during influenza infection

Studies have shown that Lcn2 plays a role in iron trafficking and has chemotactic and bacteriostatic properties^25–27^. It has also been used as a biomarker for acute kidney injury because of its release due to tubular damage. Recently, it has been shown that Lcn2 is induced in coronary heart diseases, heart failure, and myocarditis and plays a role in the pathophysiology of cardiovascular diseases (CVD)^25,29^. We also found that Stat1^−/−^ showed increased expression of Lcn2 when compared to WT mice (Fig. 4A,B). However, it is not clear that the influenza infection itself induces Lcn2 in the heart. To answer that we analyzed the expression of Lcn2 in WT mice at different time points either infected with PBS or influenza. We found that influenza infection induces the levels of Lcn2 in the heart on day 3 (early) and day 7 (peak) of influenza infection (Supplementary Fig. 5). We also found the levels returned to basal level on day 14 (recovery phase) of influenza infection (Supplementary Fig. 5). These data suggest that the presence of the virus induces Lcn2 during the early and peak phases of influenza infection.

Next, we analyzed whether the Lcn2 influences the frequency of neutrophils in the heart during influenza infection. We found that there was no significant difference in CD45+ CD11b+ Ly6G+ neutrophils present in the hearts between WT or Lcn2^−/−^ mice infected with influenza (Supplementary Fig. 6A,B). These data suggest that the Lcn2 induction was not directly correlated with the frequency of neutrophils.

**Figure 5:**
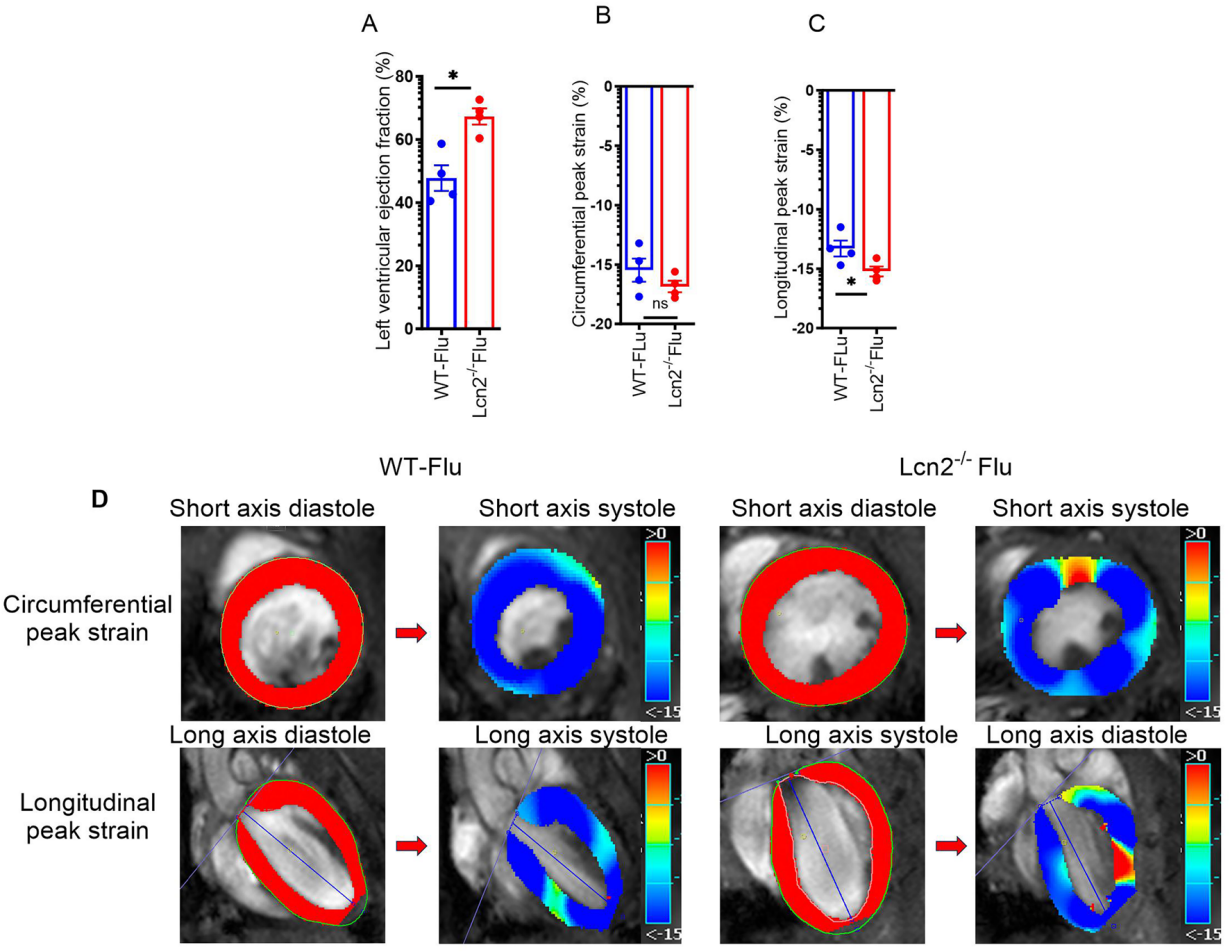
The elimination of Lcn2 expression improves heart functions during influenza infection. WT and Lcn2^−/−^ mice were treated with PBS or 10^3^ PFU of influenza A PR/8/34 on day 0 and left for 7 days. The percentage of left ventricle ejection fraction (LVEF) (n = 4) (**A**), percentage of circumferential peak strain (n = 4) (**B**), and percentage of longitudinal peak strain (n = 4) (**C**) were analyzed from micro-MRI live imaging from C57BL/6 mice treated with PBS or influenza. MRI images of LVEF, and circumferential and longitudinal peak strains of the heart samples from WT and Lcn2^−/−^ mice infected with influenza (**D**). The top row shows circumferential strain overlayed on the short-axis mid-slice cardiac cycle. The bottom row shows longitudinal strain overlayed on the long axis 4 chamber cardiac cycle. The blue line represents the long axis extent that runs from the aorta to the apex of the heart to calculate the LV function parameters (**D**). Data are represented as mean ± SEM. Significance was tested by unpaired t-test or one-way ANOVA. *p < 0.05, **p < 0.01, *ns* not significant. Each experiment was independently performed two or more times, and the representative data is shown.

Next, we conducted an MRI scan to determine whether the Lcn2 affects heart functions. We found that Lcn2^−/−^ mice infected with influenza had an increased LVEF compared to WT mice infected with influenza (Fig. 5A). The LVEF of the Lcn2^−/−^ mice was almost on par with WT mice treated with PBS from our initial MRI study. The circumferential peak strain and longitudinal peak strain were not different between WT and Lcn2^−/−^ mice (Fig. 5B–D). These data suggest that Lcn2 has a pathogenic role in inflammatory damage to the heart, resulting in decreased function. In the absence of Lcn2 heart function improves in Lcn2^−/−^ mice when compared to WT mice.

Overall, our data suggest that influenza infection increases neutrophil and lymphocytic recruitment in the heart and affects the LVEF during influenza-induced myocarditis. Further, our data suggest that STAT1 suppresses neutrophilic infiltration and the induction of Lcn2 in the heart, and Lcn2 affects the LVEF in influenza-induced myocarditis.

## Discussion

During influenza infection, type I, type II, and type III interferons (IFNs) and their downstream signaling events are crucial for the control of viral infection^15,16,32,33^. These IFNs and inflammatory mediators recruit monocytes and neutrophils to the lungs to help control the viral infection. The direct antiviral effect is mediated through the induction of interferon-stimulated genes. In our study, we found increased expression of Stat1, Stat2, Mx1, Oasl2, and ISG15 in the heart during pulmonary influenza infection. These data suggest that the influenza infection induces interferon responses in the heart.

Viral infection induces a variety of proinflammatory cytokines and chemokines including IL-1b, IL-6, TNFa, CCL2, CCL3, CXCL1, CXCL2, CXCL9 and CXCL10^34,35^. These cytokines and chemokines recruit innate immune cells such as neutrophils, macrophages, and NK cells to control the virus^35,36^. In our study, we found increased expression of CCL2, CCL3, CXCL9, and CXCL10 in the heart following PR8, pH1N1, or H3N2 strains of influenza infection. These data suggest that the influenza infection in the heart induces these chemokines to recruit the innate immune cells such as neutrophils and macrophages to control the virus. In our study, we found increased neutrophils and CD4+ T cells in the heart in response to influenza infection suggesting that there is cellular recruitment to control the virus. Similarly, previous studies have shown the presence of cardiomyocyte degeneration, and macrophage and lymphocyte infiltration into the heart during influenza infection^37^.

In our study, we found the virus in the heart on days 4 and 7 post-influenza infection. These data suggest that the virus infects the heart directly.

We next found a decreased LVEF and increased longitudinal peak strain in influenza-infected mice when compared to PBS-treated mice. Previous studies have shown that both the systolic and diastolic functions of the left ventricle were impaired in response to influenza infection^37^. Our study further shows an increase in the longitudinal peak strain in response to influenza infection. These data suggest that normal heart function is compromised due to influenza infection.

Our previous studies suggested that STAT1 regulates Th17 response during influenza infection^30^. In our study, we found no differences in the expression of IL-17F, IL-17RA, IL-22RA1, and IL-22RA2 and the levels of IL-17A in the heart in between WT and Stat1^−/−^ mice. However, we found increased levels of Lcn2 in Stat1^−/−^ mice hearts when compared to WT controls. Studies also have shown that IL-17 induces Lcn2^31,38^. These data suggest that Stat1 plays a role in regulating the expression of Lcn2 independent of Th17 responses. IL-17 is known to drive the induction of neutrophil-attracting chemokines such as CXCL1 and GCSF to mediate granulopoiesis and neutrophil recruitment to the mucosal sites^39^. In our study, we found increased neutrophils in Stat1^−/−^ mice suggesting that Stat1 regulates neutrophilic infiltration into the heart. This may be because of IL-17 in the circulation or lung thereby increasing neutrophils in the heart.

As previously mentioned Lcn2 is shown to be induced in coronary heart diseases, heart failure, and myocarditis and plays a role in the pathophysiology of CVD^25,29^. In our study, we found that LVEF is preserved in the absence of Lcn2 suggesting that Lcn2 plays a crucial role in the pathology of influenza-induced myocarditis. However, no significant differences were observed in circumferential and longitudinal peak strain between WT and Lcn2^−/−^ mice suggesting that there is a possible mild protective effect in the absence of Lcn2 in the heart during the influenza infection. In that case, an Lcn2 inhibitor is likely to be useful therapeutically to overcome the pathological effects caused by influenza-induced myocarditis. Previously studies have shown that Lcn2 is responsible for increasing iron accumulation within cardiomyocytes of a failing myocardium leading to cardiomyocyte apoptosis^40^. Our data suggest that in the absence of Lcn2 expression, there is likely decreased iron accumulation within the influenza-infected cardiomyocytes resulting in decreased apoptosis and preserved LVEF. Conversely, in our data showing an increase in Lcn2 expression in the absence of Stat1 expression, there is a possible increase in iron accumulation within the virally infected cardiomyocytes which subsequently increases apoptosis of the virally infected cardiomyocytes. Overall, our data suggest that Stat1 suppresses the neutrophilic infiltration and the induction of Lcn2 thereby suppressing the Lcn2-mediated pathological effects during influenza-induced myocarditis.

## Materials and methods

### Mice

WT C57BL/6 (6–8-week-old) and Lcn2^−/−^ mice were purchased from Taconic Farms (Germantown, NY). Stat1^*−/−*^ mice were obtained from Dr. Christian Schindler (Columbia University) and Dr. David Levy (New York University). Lcn2^−/−^ mice were purchased from Jackson Laboratory. Stat1^−/−^ and Lcn2^−/−^ mice in C57BL/6 background were bred and maintained under specific pathogen-free conditions at the animal facility at UPMC Children’s Hospital of Pittsburgh. In vivo studies were performed on age-matched adult male or female mice. All experiments were approved by the University of Pittsburgh IACUC (Protocol number 23063262). All the authors complied with the IACUC guidelines, and the study is reported in accordance with ARRIVE guidelines.

### Influenza A/PR/8/34 H1N1 infection

Influenza virus A/Puerto Rico/8/1934 (H1N1) virus was propagated by using Madin-Darby canine kidney (MDCK) cells. The cells were maintained in DMEM with 10% FBS (Bio-Techne, Minneapolis, MN). The cells were washed with PBS, and infected 0.001 MOI of influenza virus A/Puerto Rico/8/1934 (H1N1) in DMEM with 0.2% bovine serum albumin (Invitrogen, Waltham, MA), and 2 μg/ml of l-tosylamido-2-phenyl ethyl chloromethyl ketone (TPCK) (Sigma–Aldrich, MO). The virus-containing supernatant was harvested after 72 h, and the viral titer was determined by standard plaque assay.

Mice were infected with 1000 pfu of Influenza A/PR/8/34 H1N1 via an oropharyngeal route of administration. Mice were incubated for 7 days, and the lungs and the heart were harvested. Viral burden from the lungs and the heart was determined by plaque assay. The gene expression levels of influenza M protein were analyzed by using the following primers. Forward: 5′-GGACTGCAGCGTAGACGCTT-3′, Reverse: 5′-CATCCTGTTGTATATGAGGCCCAT-3′, Probe: 5′-/56-FAM/CTCAG TTAT/ZEN/TCTGCTGGTGCACTTGCCA/3IABkFQ/-3′.

### Quantitative real-time PCR

RNA was isolated by using the TRIzol reagent and its associated method as per the manufacturer’s directions (Thermo Fisher, Waltham, MA). The isolated RNA was quantified and converted into cDNA using an iScript cDNA synthesis kit (Bio-Rad, Hercules, CA). Gene expression levels were measured by RT-PCR using Assay-on-demand Taqman primers and probes from Thermo Fisher Scientific (Waltham, MA) and Integrated DNA Technologies (Coralville, IA).

### Flow cytometry

Mouse hearts were perfused with 20 ml of ice-cold PBS through the right and left ventricles and collected in RPMI media. Then the hearts were chopped into small pieces (~ 2 mm) and digested using 10 U of DNAse and 300 U collagenase II (Sigma, St. Louis, MO) in RPMI media for 1 h in a 37 °C shaking incubator. After one hour the tissues were passed through a 70 μM filter to isolate single cells. The RBC was lysed using RBC lysis buffer (Thermo Fisher Scientific, Waltham, MA). The single cells were stained with CD45 (30-F11, leukocyte common antigen), CD11b (M1/70, monocytes), Gr-1 (RB6-8C5, neutrophils) antibodies, and the data were analyzed using Flow jo analysis software.

### In-vivo cardiac MRI

Anesthesia induction was achieved by using isoflurane liquid (Piramal Pharma Ltd) for inhalation, which is vaporized using a SurgiVet Isotec (VetEquip) vaporizer. The percentage of isoflurane to oxygen is 4% to initially anesthetize the mouse inside an induction chamber. The depth of anesthesia was monitored by toe reflex, extension of limbs, and spine positioning. Once the plane of anesthesia was established, the mouse was placed on a designated animal bed for imaging and the anesthesia was maintained by 1.0 to 1.5% isoflurane with 100% oxygen via a nose cone. The body temperature was monitored by an optical rectal temperature probe which is used for feedback control of a warm air blower around the animal to maintain the core temperature 37 °C ± 0.5 °C. Respiration was continuously monitored by placing a small pneumatic pillow under the animal’s diaphragm which was connected to a magnet-comparable pressure transducer feeding to a physiological monitoring computer equipped with respiration-waveform measuring software (SA Instruments, Stony Brook, NY). The respiration waveform was automatically processed to detect the inspiration, expiration, and respiration rates. In-vivo cardiac MRI (CMR) was carried out on a Bruker Biospec 7T/30 system (Bruker Biospin MRI, Billerica, MA) with the 35 mm quadrature coil for both transmission and reception. Free-breathing-no-gating cine MRI with retrospective navigators was acquired with the Bruker Intragate module. Multi-planar cine movies covering the entire heart volumes with 20 cardiac phases were acquired for short-axis and long-axis 4-chamber views with the following parameters: Field of view (FOV) = 2.5 cm × 2.5 cm, slice thickness = 1 mm for long-axis views and 1.2 mm for short-axis views, inter-slice gap = 0, acquisition matrix = 256 × 256, in-plane resolution = 98 μm, flip angle (FA) = 10 degrees, echo time (TE) = 3.059 ms, repetition time (TR) = 5.653 ms, number of repetition = 300, total scan time = 3 min 39 s. Systolic functions and strains were analyzed with the FDA-approved Circles Cvi42 software. The left ventricular endocardium and epicardium boundaries of each imaging slice at the end-systole (ES) and the diastole (ED) were defined by a blinded operator.

### Measurement of Lipocalin 2 protein

Hearts were homogenized using 1 ml PBS, and the supernatants were collected and stored in a − 80 °C freezer. Lipocalin protein was analyzed using an ELISA kit (R&D Systems, Minneapolis, MN).

### Cardiomyocyte isolation from adult mouse hearts

Primary adult mouse cardiomyocytes were isolated from WT mice as described before^41^. The myocardium was perfused with EDTA and perfusion buffers to remove blood and subsequently digested using a collagenase buffer containing collagenases II and IV, and protease XIV (Sigma, St. Louis, MO). Cells were filtered using 100 μM filters and plated at roughly 25,000 cells per well. The cell culture plates were coated with 5 μg/ml mouse laminin (Thermo Fisher Scientific, Waltham, MA), and left to incubate in a 37 °C CO2 incubator for one hour, after which they were washed with PBS. Cardiomyocytes were cultured with M199 media supplemented with 10 mM 2,3-butanedione monoxime (BDM), 100 units/ml penicillin with 100 ug/ml streptomycin, 0.1% w/v bovine serum albumin, insulin-transferrin-selenium (ITS) supplement, and a chemically defined (CD) lipid supplement (Thermo Fisher Scientific, Waltham, MA). Cells were treated with this media and or influenza, for 24 h, and collected for gene expression analysis.

### Statistical analysis

All the data are presented as mean ± SEM. Significance was tested by students t-test or a one-way ANOVA followed by Tukey’s posthoc test. All the statistical analyses were performed by GraphPad Prism software.

### Supplementary Information

Supplementary Figures.

## Data Availability

The datasets used and/or analyzed during the current study are available from the corresponding author on reasonable request.

## References

[1] Nguyen JL (2016). Seasonal influenza infections and cardiovascular disease mortality. JAMA Cardiol..

[2] Warren-Gash C, Smeeth L, Hayward AC (2009). Influenza as a trigger for acute myocardial infarction or death from cardiovascular disease: A systematic review. Lancet Infect. Dis..

[3] Collins SD (1932). Excess mortality from causes other than influenza and pneumonia during influenza epidemics. Public Health Rep..

[4] Madjid M (2007). Influenza epidemics and acute respiratory disease activity are associated with a surge in autopsy-confirmed coronary heart disease death: Results from 8 years of autopsies in 34,892 subjects. Eur. Heart J..

[5] Ludwig A, Lucero-Obusan C, Schirmer P, Winston C, Holodniy M (2015). Acute cardiac injury events ≤30 days after laboratory-confirmed influenza virus infection among U.S. veterans, 2010–2012. BMC Cardiovasc. Disord..

[6] Mamas MA, Fraser D, Neyses L (2008). Cardiovascular manifestations associated with influenza virus infection. Int. J. Cardiol..

[7] Ona MA (2012). A case of fatal fulminant myocarditis presenting as an acute ST-segment elevation myocardial infarction and persistent ventricular tachyarrhythmia associated with influenza A (H1N1) virus in a previously healthy pregnant woman. Cardiology.

[8] Dawood FS (2012). Estimated global mortality associated with the first 12 months of 2009 pandemic influenza A H1N1 virus circulation: A modelling study. Lancet Infect. Dis..

[9] Barbandi M (2012). A case series of reversible acute cardiomyopathy associated with H1N1 influenza infection. Methodist Debakey Cardiovasc. J..

[10] Bratincsak A (2010). Fulminant myocarditis associated with pandemic H1N1 influenza A virus in children. J. Am. Coll. Cardiol..

[11] Baruteau AE, Boimond N, Ramful D (2010). Myocarditis associated with 2009 influenza A (H1N1) virus in children. Cardiol. Young.

[12] Al-Amoodi M (2010). Fulminant myocarditis due to H1N1 influenza. Circ. Heart Fail..

[13] Rezkalla SH, Kloner RA (2010). Influenza-related viral myocarditis. WMJ.

[14] Iwasaki A, Pillai PS (2014). Innate immunity to influenza virus infection. Nat. Rev. Immunol..

[15] Durbin RK, Kotenko SV, Durbin JE (2013). Interferon induction and function at the mucosal surface. Immunol. Rev..

[16] Garcia-Sastre A (1998). The role of interferon in influenza virus tissue tropism. J. Virol..

[17] Damjanovic D, Small CL, Jeyanathan M, McCormick S, Xing Z (2012). Immunopathology in influenza virus infection: Uncoupling the friend from foe. Clin. Immunol..

[18] Lee AJ, Ashkar AA (2018). The dual nature of type I and type II interferons. Front. Immunol..

[19] Giamarellos-Bourboulis EJ (2020). Complex immune dysregulation in COVID-19 patients with severe respiratory failure. Cell Host Microbe.

[20] Huang C (2020). Clinical features of patients infected with 2019 novel coronavirus in Wuhan, China. Lancet.

[21] Zhou F (2020). Clinical course and risk factors for mortality of adult inpatients with COVID-19 in Wuhan, China: A retrospective cohort study. Lancet.

[22] Kido H (2012). Role of host cellular proteases in the pathogenesis of influenza and influenza-induced multiple organ failure. Biochim. Biophys. Acta.

[23] Pan HY (2011). Up-regulation of ectopic trypsins in the myocardium by influenza A virus infection triggers acute myocarditis. Cardiovasc. Res..

[24] Filgueiras-Rama D (2020). Human Influenza A virus causes myocardial and cardiac-specific conduction system infection associated with early inflammation and premature death. Cardiovasc. Res..

[25] Romejko K, Markowska M, Niemczyk S (2023). The review of current knowledge on neutrophil gelatinase-associated lipocalin (NGAL). Int. J. Mol. Sci..

[26] Schmidt-Ott KM (2007). Dual action of neutrophil gelatinase-associated lipocalin. J. Am. Soc. Nephrol..

[27] Gupta K, Shukla M, Cowland JB, Malemud CJ, Haqqi TM (2007). Neutrophil gelatinase-associated lipocalin is expressed in osteoarthritis and forms a complex with matrix metalloproteinase 9. Arthritis Rheum..

[28] Awadalla M (2019). Influenza vaccination and myocarditis among patients receiving immune checkpoint inhibitors. J. Immunother. Cancer.

[29] Cruz DN, Gaiao S, Maisel A, Ronco C, Devarajan P (2012). Neutrophil gelatinase-associated lipocalin as a biomarker of cardiovascular disease: A systematic review. Clin. Chem. Lab. Med..

[30] BenjaminLee RG, Manni ML, McHugh KJ, Mandalapu S, Robinson KM, Alcorn JF (2017). STAT1 is required for suppression of type 17 immunity during influenza and bacterial superinfection. ImmunoHorizons.

[31] Robinson KM (2014). Influenza A virus exacerbates Staphylococcus aureus pneumonia in mice by attenuating antimicrobial peptide production. J. Infect. Dis..

[32] Durbin JE (2000). Type I IFN modulates innate and specific antiviral immunity. J. Immunol..

[33] Jewell NA (2010). Lambda interferon is the predominant interferon induced by influenza A virus infection in vivo. J. Virol..

[34] Sanders CJ, Doherty PC, Thomas PG (2011). Respiratory epithelial cells in innate immunity to influenza virus infection. Cell Tissue Res..

[35] Kohlmeier JE, Woodland DL (2009). Immunity to respiratory viruses. Annu. Rev. Immunol..

[36] Guo L (2017). Pulmonary immune cells and inflammatory cytokine dysregulation are associated with mortality of IL-1R1 (−/−)mice infected with influenza virus (H1N1). Zool. Res..

[37] Kotaka M, Kitaura Y, Deguchi H, Kawamura K (1990). Experimental influenza A virus myocarditis in mice. Light and electron microscopic, virologic, and hemodynamic study. Am. J. Pathol..

[38] Ferreira MC (2014). Interleukin-17-induced protein lipocalin 2 is dispensable for immunity to oral candidiasis. Infect. Immun..

[39] Aujla SJ, Dubin PJ, Kolls JK (2007). Th17 cells and mucosal host defense. Semin. Immunol..

[40] Xu G (2012). Lipocalin-2 induces cardiomyocyte apoptosis by increasing intracellular iron accumulation. J. Biol. Chem..

[41] Ackers-Johnson M, Foo RS (2019). Langendorff-free isolation and propagation of adult mouse cardiomyocytes. Methods Mol. Biol..

