# Supplementary material for: STAT1 regulates neutrophil gelatinase B-associated lipocalin induction in influenza-induced myocarditis
Source: Sci Rep. 2024 May 15;14:11124. doi: 10.1038/s41598-024-61953-z (PMC11096373; doi:10.1038/s41598-024-61953-z)
Supplement: Supplementary file 1 — Supplementary Figures. [file 41598_2024_61953_MOESM1_ESM.docx]

**
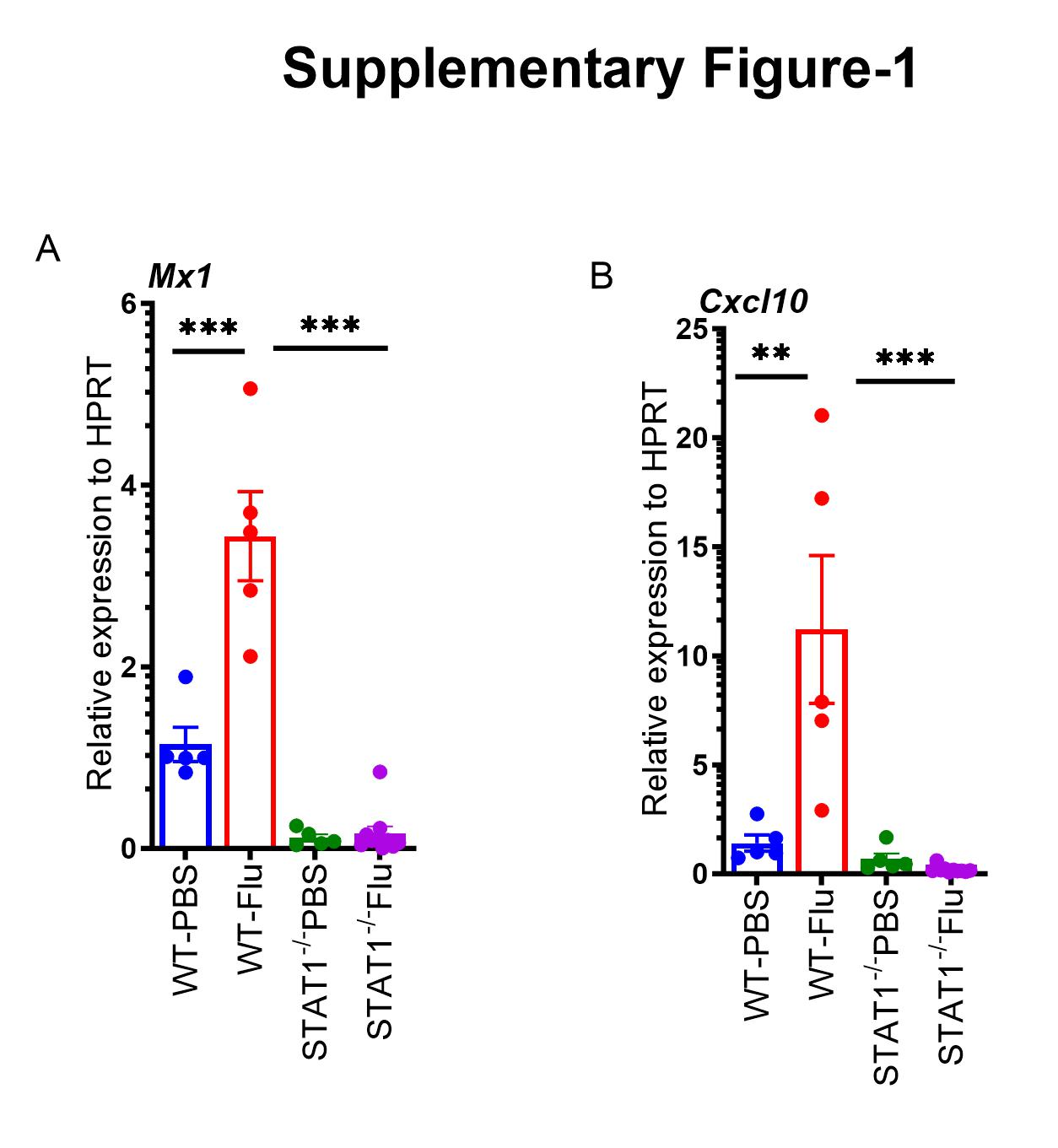
**

**Supplementary Figure-1 The elimination of STAT1 suppresses the expression of ISGs during influenza infection.**

WT or STAT1^-/-^ mice were treated with PBS or 10^3^ PFU of influenza A PR/8/34 on day 0 and left for 7 days. Relative expression of Mx1 and CXCL10 compared between B6 and STAT1^-/-^ mice under the same conditions (n=5-10) (A&B). Data are represented as mean±SEM. Significance was tested by unpaired t-test or one-way ANOVA. *p < 0.05, **p < 0.01, ns, not significant. Each experiment was independently performed two or more times, and the representative data is shown.

**
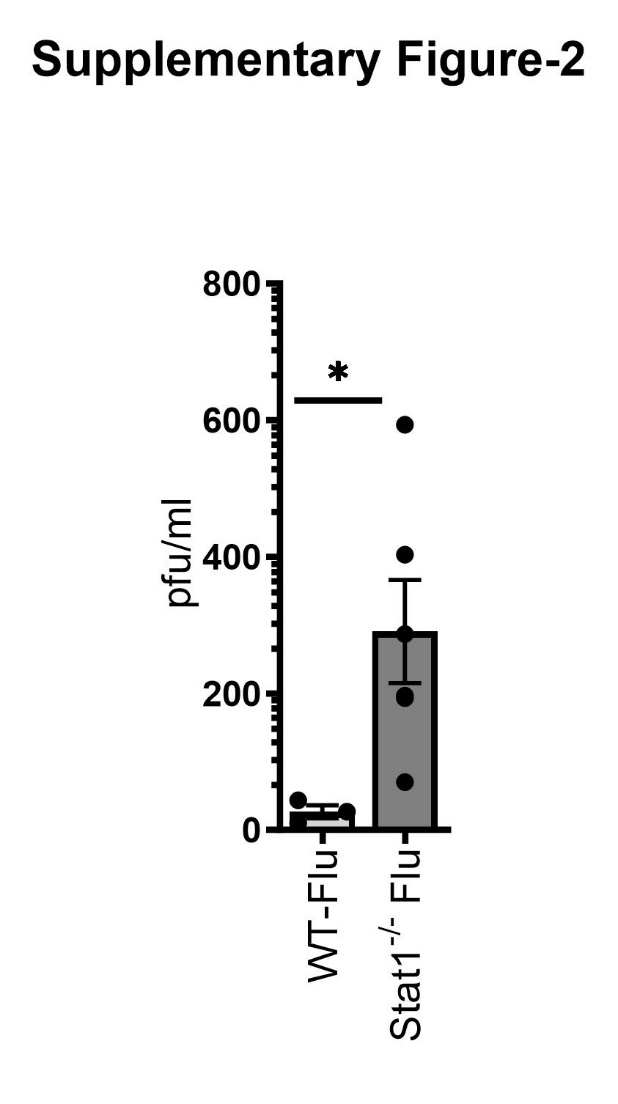
**

**Supplementary Figure-2 The elimination of STAT1 impairs viral control during influenza infection.**

WT or STAT1^-/-^ mice were treated with 10^3^ PFU of influenza A PR/8/34 on day 0 and left for 7 days. Plaque assay was performed from the heart homogenates from WT and Stat1^-/-^ mice (n=3-6). Data are represented as mean±SEM. Significance was tested by unpaired t-test. *p < 0.05, **p < 0.01, ns, not significant. Each experiment was independently performed two or more times, and the representative data is shown.

**
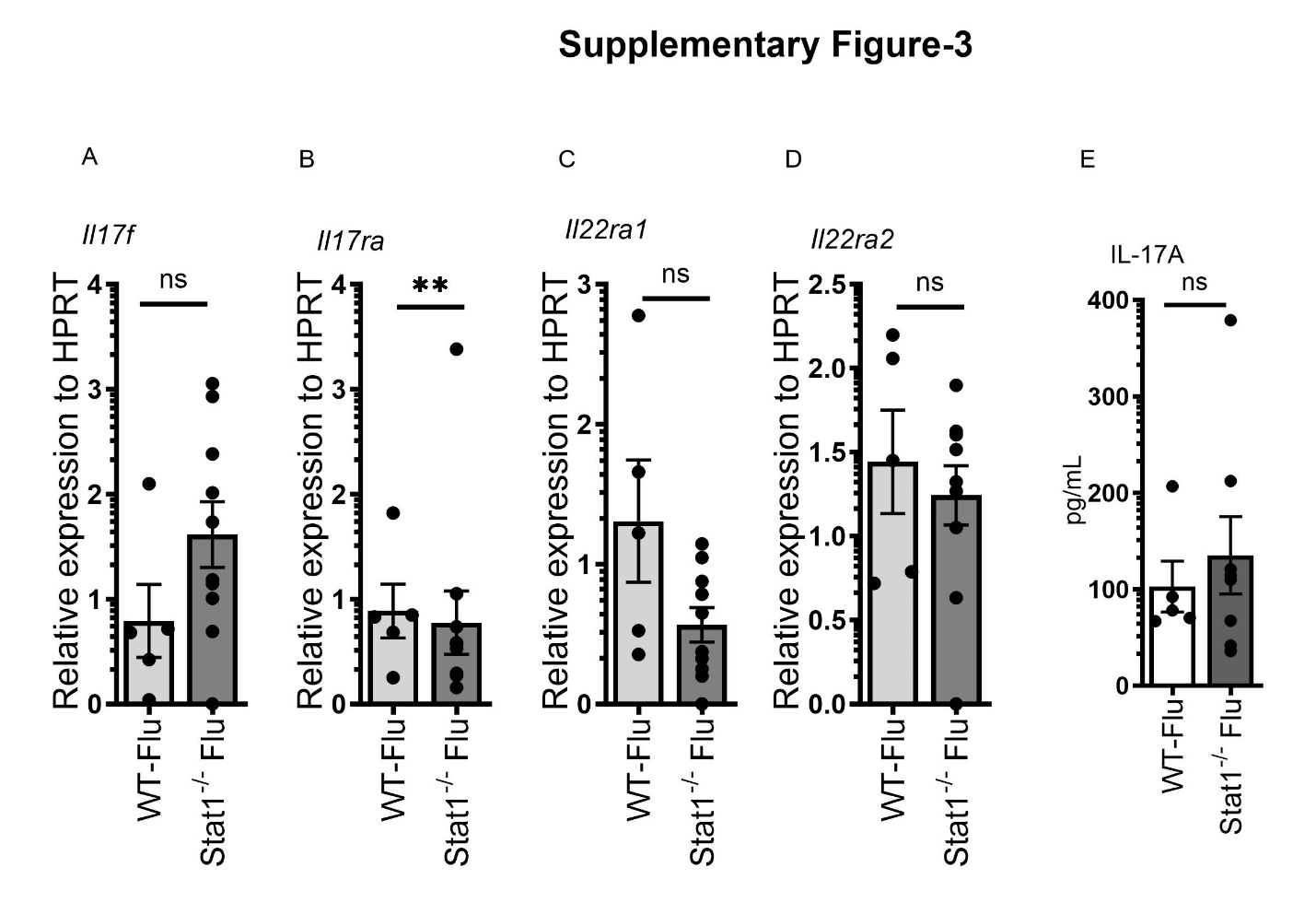
Supplementary Figure-3 The elimination of STAT1 expression does not impact the expression of IL-17 and IL-22 responses during influenza infection.**

WT or STAT1^-/-^ mice were treated with 10^3^ PFU of influenza A PR/8/34 on day 0 and left for 7 days. Relative expressions of IL-17RA, IL-17F, IL-22RA1, and IL-22RA2 were analyzed from C57BL/6 and STAT1^-/-^ mice hearts (n=5-10) (A-D). The levels of IL-17A were analyzed from WT and Stat1^-/-^ mice hearts (n=5-8) (E). Data are represented as mean±SEM. Significance was tested by unpaired t-test or one-way ANOVA. *p < 0.05, **p < 0.01, ns, not significant. Each experiment was independently performed two or more times, and the representative data is shown.

**
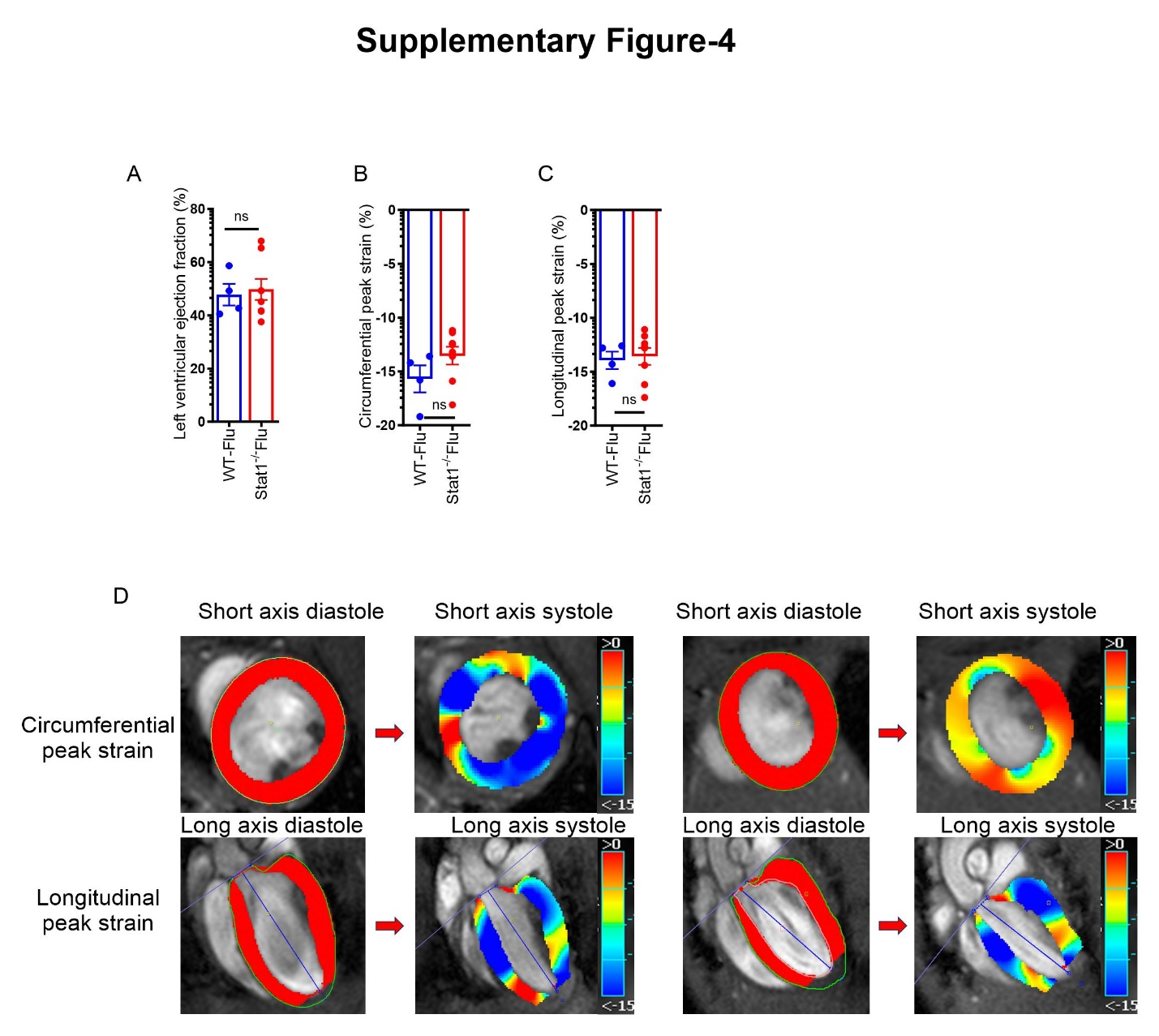
Supplementary Figure-4. The elimination of STAT1 expression does not impact the heart functions during influenza infection.**

WT or STAT1^-/-^ mice were treated with 10^3^ PFU of influenza A PR/8/34 on day 0 and left for 7 days. The percentage of left ventricle ejection fraction (LVEF) (8=11) (A), percentage of circumferential strain (8-11) (B), and percentage of longitudinal strain (n=8-11) (C) were analyzed from MRI live imaging from WT and Stat1^-/-^ mice treated with influenza. The representative images are shown (D). The top row shows circumferential strain overlayed on the short-axis mid-slice cardiac cycle. The bottom row shows longitudinal strain overlayed on the long-axis 4-chamber cardiac cycle. The blue line represents the long axis extent that runs from the aorta to the apex of the heart to calculate the LV function parameters (D). Data are represented as mean±SEM. Significance was tested by unpaired t-test or one-way ANOVA. *p < 0.05, **p < 0.01, ns, not significant. Each experiment was independently performed two or more times, and the representative data is shown.

**
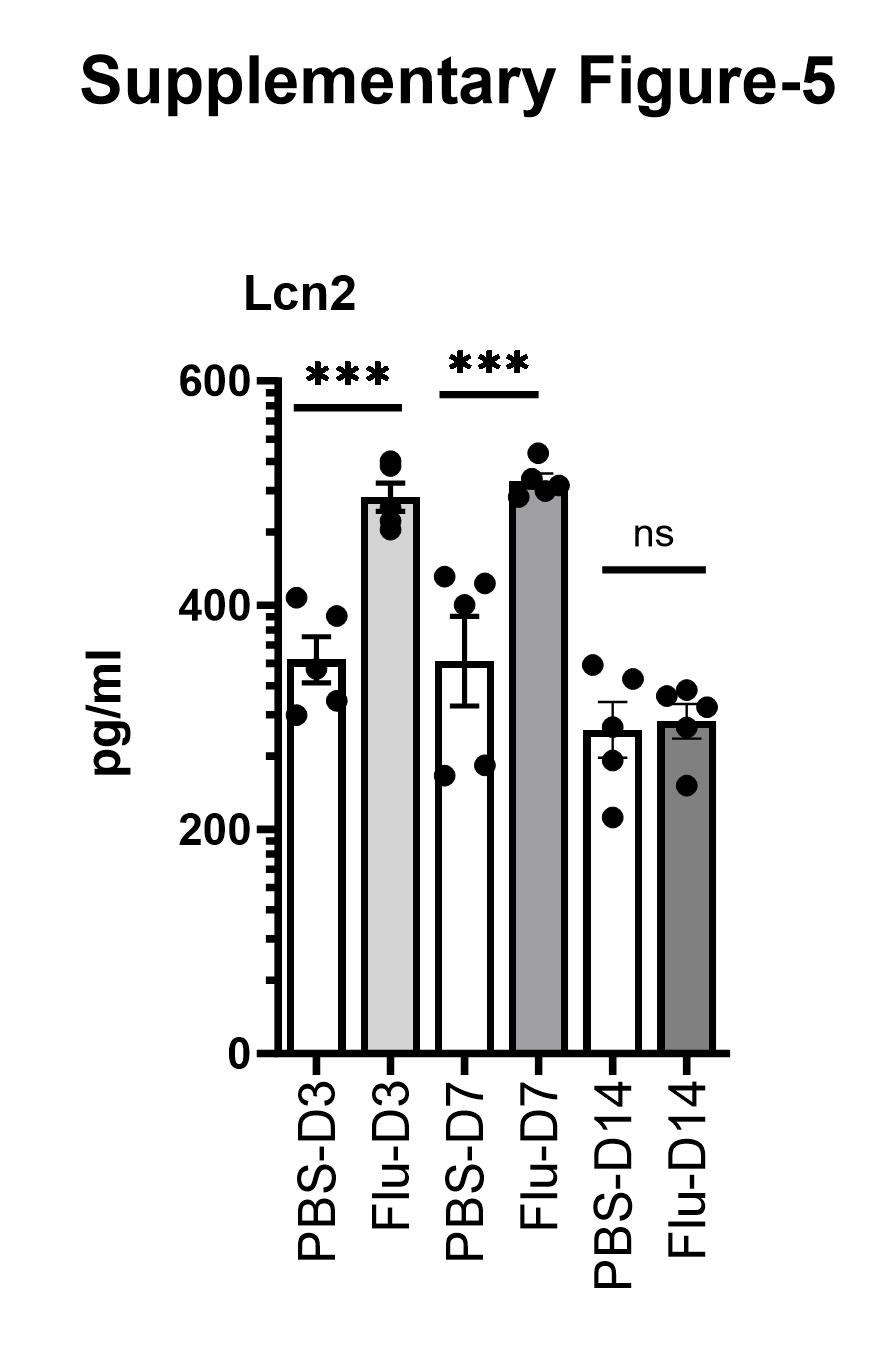
**

**Supplementary Figure-5. Lcn2 is induced in response to influenza infection.**

C57BL/6 mice were treated with PBS or 10^3^ PFU of influenza A PR/8/34 on day 0 and left for 3, 7, or 14 days. Levels of Lcn2 protein were measured via ELISA from heart homogenates collected from mice at each of the three time points (n=5). Data are represented as mean±SEM. Significance was tested by unpaired t-test or one-way ANOVA. *p < 0.05, **p < 0.01, ns, not significant. Each experiment was independently performed two or more times, and the representative data is shown.

**
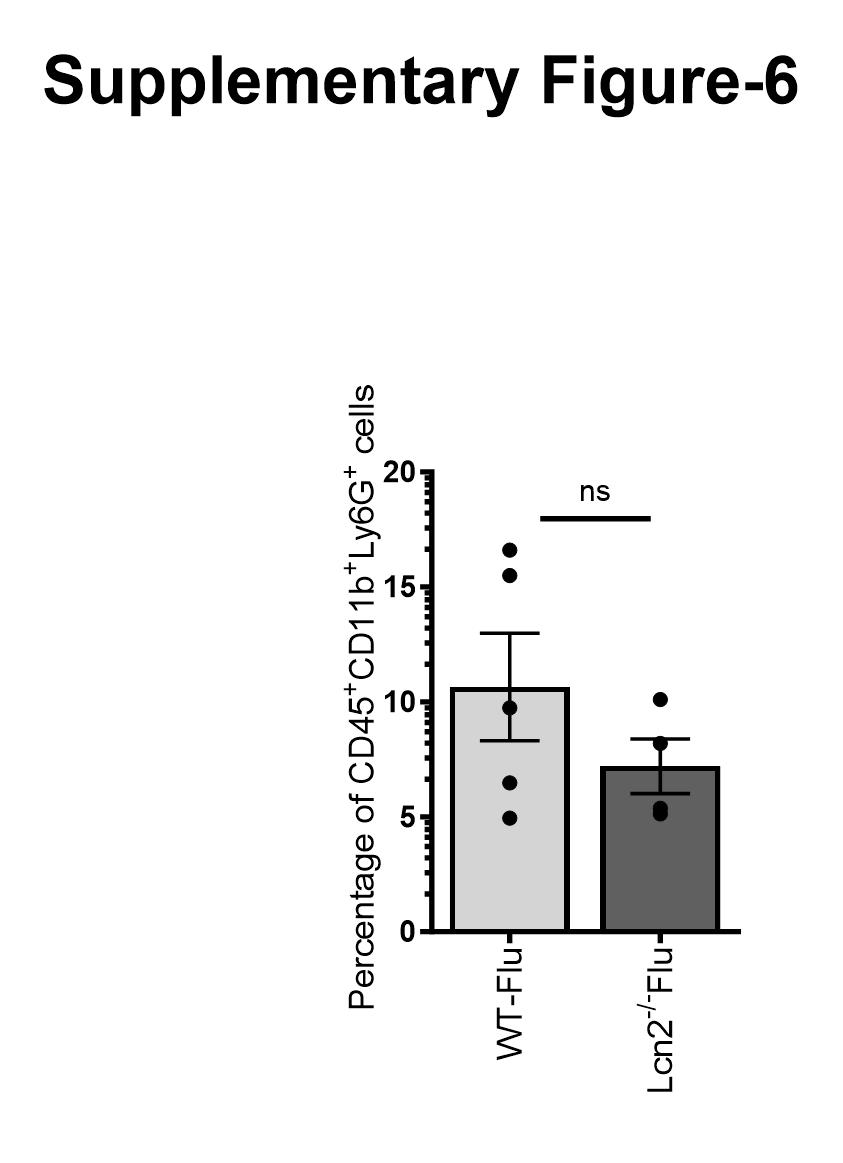
**

**Supplementary Figure-6. The frequency of neutrophils is not compromised in the absence of Lcn2 during influenza infection.**

WT or Lcn2^-/-^ mice were treated with 10^3^ PFU of influenza A PR/8/34 on day 0 and left for 7 days. The percentage of CD45^+^CD11b^+^LyG^+^ cells in the heart was measured via flow cytometry (n=4-5) (A). Data are represented as mean±SEM. Significance was tested by unpaired t-test or one-way ANOVA. *p < 0.05, **p < 0.01, ns, not significant. Each experiment was independently performed two or more times, and the representative data is shown.
